# Metabolic Syndrome and Schizophrenia: Adding a Piece to the Interplay Between the Kynurenine Pathway and Inflammation

**DOI:** 10.3390/metabo15030176

**Published:** 2025-03-05

**Authors:** Jacopo Sapienza, Giulia Agostoni, Federica Repaci, Marco Spangaro, Stefano Comai, Marta Bosia

**Affiliations:** 1 Schizophrenia Research and Clinical Unit, IRCCS San Raffaele Scientific Institute, 20127 Milan, Italy; sapienza.jacopo@hsr.it (J.S.);; 2 Department of Humanities and Life Sciences, University School for Advanced Studies IUSS, 27100 Pavia, Italy; 3 Department of Pharmaceutical and Pharmacological Sciences, University of Padua, 35123 Padua, Italy; 4 Division of Neurosciences, IRCCS San Raffaele Scientific Institute, 20132 Milan, Italy; 5 Department of Psychiatry, McGill University, Montreal, QC H3A 0G4, Canada; 6 Department of Biomedical Sciences, University of Padua, 35123 Padua, Italy; 7 School of Medicine, Vita-Salute San Raffaele University, 20132 Milan, Italy

**Keywords:** kynurenic acid (KYNA), treatment-resistant schizophrenia (TRS), cognition

## Abstract

The biology of schizophrenia is highly complex and multifaceted. Numerous efforts have been made over the years to disentangle the heterogeneity of the disease, gradually leading to a more detailed understanding of its underlying pathogenic mechanisms. Two cardinal elements in the pathophysiology of schizophrenia are neuroinflammation and alterations of neurotransmission. The kynurenine (KYN) pathway (KP) is of particular importance because it is inducted by systemic low-grade inflammation in peripheral tissues, producing metabolites that are neuroactive (i.e., modulating glutamatergic and cholinergic neurotransmission), neuroprotective, or neurotoxic. Consequently, the KP is at the crossroads between two primary systems involved in the pathogenesis of schizophrenia. It bridges the central nervous system (CNS) and the periphery, as KP metabolites can cross the blood–brain barrier and modulate neuronal activity. Metabolic syndrome plays a crucial role in this context, as it frequently co-occurs with schizophrenia, contributing to a sub-inflammatory state able to activate the KP. This narrative review provides valuable insights into these complex interactions, offering a framework for developing targeted therapeutic interventions or precision psychiatry approaches of the disorder.

## 1. Introduction

Schizophrenia is a complex and debilitating psychiatric disorder characterized by a range of symptoms, including perceptual and cognitive disturbances, emotional flattering, negative symptoms, and altered behaviors [1]. The heterogeneity of symptoms probably lies in the biological variability that characterizes schizophrenia, making understanding its neurobiological underpinnings essential for developing effective therapeutic interventions. In the last decade, the intricate interplay between metabolic syndrome (MetS), neuroinflammation, and the kynurenine (KYN) pathway (KP) has attracted considerable attention in the pathophysiology of schizophrenia [2,3,4,5,6,7,8].

The Clinical Antipsychotic Trials of Intervention Effectiveness (CATIE) revealed that individuals with schizophrenia are more likely to develop MetS compared to the general population, with a prevalence exceeding 40% [9]. This vulnerability likely arises from multiple factors, including antipsychotic medications, lifestyle habits, and obesity, which contribute to higher rates of glucose intolerance, hyperglycemia, and hypertension. Notably, MetS is associated with increased peripheral levels of cytokines and chemokines and thus with a pattern of systemic sub-inflammation, predominantly due to insulin resistance caused by resistine, a hormone released by adipose tissue [4,9,10,11,12]. This systemic low-grade inflammation can induce or exacerbate neuroinflammation, a typical feature widely reported in patients with schizophrenia [6,13,14,15].

One consequence of increased synthesis of pro-inflammatory cytokines is their capacity to induce the KP, which catabolizes tryptophan (Trp) into various metabolites, including nicotinamide adenine dinucleotide (NAD+), which is essential for cellular energy production [16,17]. These metabolites exhibit diverse properties, including the modulation of cholinergic and glutamatergic neurotransmission, neuroprotection, and neurotoxicity. Among these metabolites, kynurenic acid (KYNA) and quinolinic acid (QUIN) are of particular interest due to their distinct neuroactive properties [16,17]. KYNA acts as a negative allosteric modulator of N-methyl-D-aspartate receptors (NMDARs), specifically at the glycine binding site, leading to reduced excitotoxicity but exacerbating glutamatergic hypofunction, a feature implicated in schizophrenia. KYNA also antagonizes α7 nicotinic acetylcholine receptors (α7nAChRs), which are involved in cognitive processes. This antagonism contributes to cognitive deficits and negative symptoms by impairing cholinergic neurotransmission.

In contrast, QUIN is an NMDAR agonist, which can counterbalance the KYNA-induced hypofunction of NMDARs but also induces excitotoxicity when present in excessive concentrations. This dual role highlights its contribution to neurotoxicity and neurodegeneration [16,17]. The interplay between these metabolites affects the glutamatergic and cholinergic systems, with KYNA primarily associated with neuroprotection and cognitive impairment, whereas QUIN is linked to neurotoxicity and excitotoxic damage [16,17]. Both metabolites collectively influence the pathophysiology of schizophrenia by modulating synaptic plasticity, excitotoxicity, and cognitive functioning.

The KP thus sits at the crossroads between inflammation and neurotransmission, impacting synaptic function and neuronal homeostasis [3,5,7]. Indeed, a recent meta-analysis on the biomarkers of the cerebrospinal fluid (CSF) in schizophrenia found increased levels of both KP metabolites and pro-inflammatory cytokines [18].

It is important to note that the systemic low-grade inflammation sustained by MetS induces the peripheral activation of the KP and, in turn, the release of neuroactive metabolites able to cross the blood–brain barrier (BBB) and worsen the severity of psychopathology and cognitive deficits [5,7,19,20,21]. Indeed, KYN readily crosses the BBB via a large neutral amino acid transporter, and its levels in the brain are influenced by peripheral inflammation and metabolic states. While the brain synthesizes KYN locally, up to 60% of KYN is derived from the periphery under normal physiological conditions. Immune activation, particularly during systemic inflammation, significantly increases peripheral KYN production, leading to elevated central levels [16,17].

**Figure 1:**
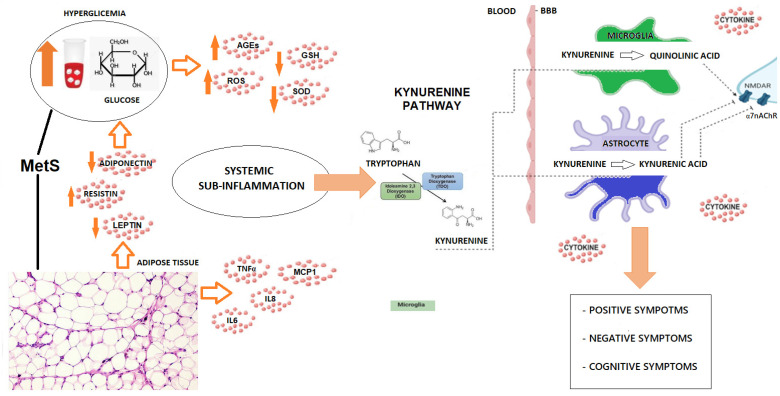
Interplay between metabolic syndrome, systemic inflammation, and kynurenine pathway. The figure depicts the contribution of hyperglycemia and visceral obesity, two components of MetS, to low-grade systemic inflammation, which, in turn, induces the KP. Higher levels of KYN in the circulation correspond to higher levels in the CNS. Then, at central level, KYN is converted into neuroactive metabolites, with implications for glutamatergic and cholinergic neurotransmission but also neurotoxicity.

This review underscores the critical involvement of the KP as a mechanistic link between MetS and schizophrenia, with a specific focus on the interplay between neuroinflammation and neurotransmission. Importantly, as shown in Figure 1, it highlights the bidirectional interaction between these systems, incorporating recent advancements in KP-associated biomarkers and evaluating their translational potential as therapeutic targets.

## 2. Metabolic Syndrome: Implications for Systemic Low-Grade Inflammation

MetS is characterized by multiple metabolic abnormalities. Standard diagnostic criteria for MetS include increased waist circumference, elevated blood pressure, high blood glucose and triglyceride levels, and reduced concentrations of high-density lipoprotein (HDL) cholesterol. The diagnosis is confirmed when three or more of these risk factors are present [22].

### 2.1. Insulin Resistance

Adipose tissue secretes various adipokines, including chemokines, cytokines, and hormones involved in energy homeostasis and inflammation [23,24]. Key pro-inflammatory mediators such as interleukin (IL)-1, IL-6, and IL-8, monocyte chemotactic protein (MCP)-1, and tumor necrosis factor (TNF)-α can induce insulin resistance [25,26,27,28]. Obesity is associated with the increased infiltration of macrophages into the adipose tissue, shifting from an anti-inflammatory M2 phenotype to a pro-inflammatory M1 phenotype [29]. This phenotypic switch results in elevated levels of pro-inflammatory cytokines and chemokines, exacerbating insulin resistance [30,31]. Furthermore, obesity-related immune dysregulation involves the upregulation of interferon (IFN)-γ + T helper type 1 cells and CD8+ T cells in adipose tissue [32]. In addition, in obese individuals, adiponectin, an insulin-sensitizing hormone, and leptin are reduced, and resistin and angiotensin are upregulated with the overall effect of worsening glucose uptake (hampering the action of insulin), hunger, and hypertension [33]. Importantly, insulin regulates glucose metabolism in the body, affecting also cognition and synaptic plasticity [34]. As a matter of fact, the nervous system can develop insulin resistance as neurons show insulin receptors and are insulin-responsive [35,36].

### 2.2. Hyperglycemia

Insulin resistance-associated hyperglycemia promotes non-enzymatic glycosylation reaction (glycation) and the tricarboxylic acid (TCA) cycle through substrate overload, causing, in turn, an imbalance in reactive oxygen species (ROS) production. In the first case, ROS are advanced glycation end-products (AGEs) generated by the Amadori compound, an intermediate metabolite of the glycation reaction [37]. AGEs, formed via non-enzymatic reactions, further propagate inflammation by binding to AGE receptors (RAGE) on immune cells [38], activating nuclear factor-κB (NFκB) signaling. This cascade induces the transcription of pro-inflammatory genes encoding cytokines, chemokines, enzymes, and growth factors [39]. On the other hand, the TCA cycle is involved in the mitochondrial electron transfer system, located on the inner side of the mitochondrial membrane. It also becomes a source of oxidative stress, as water molecules generated by the deoxidation of the electrons of oxygen molecules lead to the production of ROS as an intermediate product (mainly superoxide anions), even under physiological conditions, but even more in the hyperglycemic state [37]. Hyperglycemia also downregulates antioxidant defenses such as superoxide dismutase (SOD), catalase, and glutathione peroxidase while increasing free radical production [40,41,42]. It is important to note that, lipid peroxidation, reduced glutathione (GSH) levels, and glucose auto-oxidation further sustain systemic inflammation [43].

## 3. Metabolic Syndrome and Schizophrenia

Patients with schizophrenia frequently present with comorbid MetS, with a prevalence exceeding 40% [9]. This association is linked to systemic low-grade inflammation driven by visceral obesity, hormonal dysregulation, and increased pro-inflammatory cytokines [4,9,10,11,12]. MetS correlates with greater cognitive impairment in schizophrenia [2,44], potentially due to enhanced neuroinflammation, oxidative stress, cytotoxicity, white matter (WM) disruption, and microglial activation [5,6,13,14,15,45,46]. All these pathogenic elements have a negative impact on the cognitive outcome. Indeed, patients with MetS exhibit lower cognitive performance across multiple cognitive domains [2,44]. Interestingly, the relationship between individual MetS components and cognitive impairment in schizophrenia is well documented [44,47], though with small effect sizes [48]. Reverse causality is possible, where cognitive deficits, but also negative symptoms, may contribute to MetS through socioeconomic disadvantage, unhealthy lifestyles, less access to healthcare, carelessness, and sedentary behavior [49]. Second-generation antipsychotics, known for inducing weight gain and metabolic abnormalities, further compound MetS risk [50]. Indeed, Mitchell and colleagues compared un-medicated patients, first-episode psychoses, and patients on stable antipsychotic treatment and found that the prevalence of MetS drastically increases if antipsychotics are prescribed [51]. The KP may mediate the link between systemic inflammation and cognitive decline through neuroactive metabolites, which are formed under inflammatory conditions [5]. Some findings suggest a relationship between MetS and pharmaco-resistance, although the causal link may be bidirectional, as refractory symptoms are typically treated with higher doses of antipsychotics, particularly clozapine, which is associated with significant weight gain, dyslipidemia, and glucose intolerance [50]. Conversely, the pro-inflammatory state induced by MetS and subsequent activation of the KP may partially explain the more severe refractory symptoms observed in treatment-resistant schizophrenia (TRS). As a matter of fact, obesity, altered metabolism, and systemic low-grade inflammation correlate with an increased KYN/Trp ratio, reflecting greater Trp catabolism through the KP and elevated levels of its metabolites [52,53,54,55,56]. Therefore, peripheral inflammation driven by the dysmetabolic state may lead to enhanced concentrations of KYN in the brain through inter-organ crosstalk involving adipose tissue, skeletal muscles, the gut, and the CNS [55,56,57].

## 4. The Kynurenine Pathway and Inflammation

The degradation of Trp through the KP is initiated by two different enzymes: tryptophan 2,3-dioxygenase (TDO) and indoleamine 2,3-dioxygenase isoform 1 and 2 (IDO1 and IDO2), which differ in tissue distribution and involvement in various pathologies [58,59]. TDO, unlike IDO, is generally stimulated by glucocorticoids but not cytokines and is the limiting enzyme of the KP in the periphery [60,61]. IDO1 is expressed in peripheral organs and is activated by viral and bacterial infections [60,61], as pro-inflammatory cytokines can induce its activity. Specifically, interferon-gamma (IFN-γ) alone [60] and tumor necrosis factor-alpha (TNF-α) (synergic effect with IFN-γ) increase the transcription of the IDO1 gene [62,63]. For this reason, IDO1 is thought to be causally related to various pathological conditions. Interestingly, while IDO2 expression in the brain is limited (with scarce evidence), IDO1 expression and enzyme activity in the brain have been widely documented through genetic, histological, and biochemical studies [64,65] and have been found in cultured neurons, astrocytes, and microglia [66]. These studies indicate that IDO1 activity in the brain is low under normal physiological conditions. IDO2 also plays a role in Trp degradation under physiological conditions [67] and is mainly expressed in neuronal cells [68]. However, IFN-γ significantly increases IDO2 expression in cultured human glioma cells [69].

### The Pivotal Role of Kynurenine

**Figure 2:**
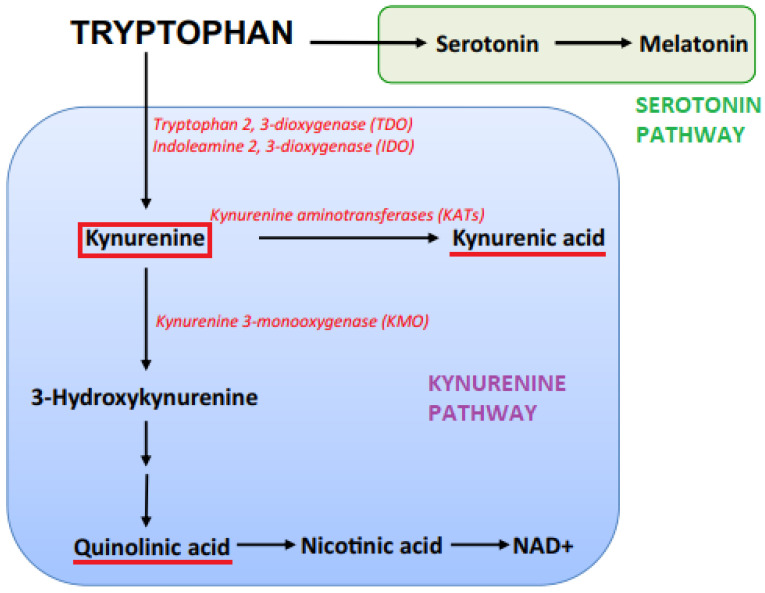
**The kynurenine pathway.** The figure represents the pivotal metabolic steps of the kynurenine pathway.

KYN [70,71] rapidly crosses the BBB via a large amino acid transporter, which can also bind 3-hydroxykynurenine (3-HKYN), and leucine [70,72]. Due to the low activity of brain TDO2, IDO1, and IDO2 compared to the periphery, 60% of brain KYN comes from the circulation. However, brain synthesis increases dramatically with immune activation [73,74], which has significant implications for schizophrenia and other mental illnesses [75,76]. In contrast, the uptake of other metabolites is limited, with passive diffusion occurring at very low rates, particularly for quinolinic acid (QUIN) and kynurenic acid (KYNA), with the latter also constrained by plasma protein binding [21]. It is important to note that the concentration of KYN in the CSF is at least 10-fold lower than in the brain tissue [77], followed by metabolism to other neuroactive compounds of the pathway [17]. For a comprehensive representation of the main metabolic steps of the pathway, see Figure 2.

## 5. The Kynurenine Pathway in Schizophrenia

There is evidence of increased KP activity in schizophrenia, as pro-inflammatory cytokines induce the IDO enzyme, leading to greater conversion of Trp into KYN [78,79]. KP activation in the brain of patients with schizophrenia has been consistently reported since the first studies over 20 years ago [80,81]. According to a recent meta-analysis by Cao and colleagues, patients have significantly higher KYN and KYNA levels compared to controls in CSF but lower plasma KYN levels, likely due to the greater transport of KYN to the CNS. Notably, KYN levels were reported to be higher after treatment with antipsychotics compared to baseline. This finding could be explained by the increased incidence of MetS due to side effects of antipsychotic drugs [76]. Moreover, Kindler and colleagues reported increased TDO expression, KYN/Trp ratio (KP activation), as well as mRNA for kynurenine aminotransferases (KATI/II) and KYNA levels only in patients with higher cytokines levels, further endorsing the essential role of both immune and KP activation in schizophrenia [8]. Concerning KYNA, elevated KYNA levels have been repeatedly observed in CSF and in postmortem studies [3,82,83]. Increased levels of KYNA in the CNS have important implications for various symptom domains, particularly for pharmacoresistant (refractory) symptoms [69,84,85].

### 5.1. Neuroactive Metabolites of the KP

Among the metabolites produced in the KP, two are of particular interest: KYNA and QUIN, due to their pharmacodynamics and their respective neuroprotective and neurotoxic potentials [7,86]. These metabolites modulate glutamatergic and cholinergic neurotransmission. KYNA acts as a negative allosteric modulator of N-methyl-D-aspartate receptor (NMDAR), blocking the glycine binding site on the NMDARs, with neuroprotective and anticonvulsant effects, but it worsens glutamatergic hypofunction, a typical feature of schizophrenia [7]. KYNA also helps reducing excitotoxicity caused by excessive glutamate release, which occurs due to the compensatory presynaptic glutamate release, a direct consequence of NMDAR hypofunction [3,87,88,89]. Another important property of KYNA is its antagonism toward α7 nicotinic acetylcholine receptors (α7nAChRs), a pool of pre-synaptic cholinergic receptors that seems to elicit pro-cognitive effects and are implicated in the pathophysiology of schizophrenia [90,91]. In contrast, QUIN is a selective NMDAR agonist, increasing NMDA stimulation and counterbalancing NMDAR hypofunction induced by KYNA. However, QUIN can also enhance excitotoxicity. Considering peripheral and central compartments, discrepancies in KYNA and QUIN levels are widely reported [76,92,93] despite higher KYNA levels in CSF, representing a robust finding according to several in vivo and postmortem studies and meta-analyses [3,16,18,76,82,83]. The decreased KYNA levels sometimes reported in the literature might be due to the predominant conversion in the CNS due to the enhanced transport of KYN into the brain, which lowers the availability of the precursor in the periphery. Interestingly, a recent postmortem study comparing the levels of KP metabolites in the brains of patients with schizophrenia and healthy controls found increased QUIN and KYNA levels in the WM but not in the gray matter of the prefrontal cortex [94], corroborating previous findings of WM abnormalities associated with KP activation [95]. Overall, KP activation has been linked to cognitive impairment and symptom severity, likely due to the combined effects of the anticholinergic and anti-glutamatergic properties of its neuroactive metabolites, along with neurotoxicity [96].

#### 5.1.1. Implications for Symptoms

NMDAR and cholinergic hypofunction have been implicated in the pathophysiology of schizophrenia and associated cognitive deficits [97]. Furthermore, NMDAR hypofunction plays an important role in the pathophysiology of TRS [7,89,98]. The NMDAR hypofunction hypothesis provides a comprehensive model of schizophrenia that integrates the dopamine hypothesis [97,99,100]. While glutamatergic abnormalities are likely involved in the pathogenesis of schizophrenia in all patients, they appear to be particularly relevant in the case of TRS [101]. Indeed, higher levels of glutamate, especially in the anterior cingulate cortex (ACC), are associated with pharmaco-resistance to non-clozapine antipsychotics, compared to patients with lower levels [98,102,103,104]. Interestingly, clozapine inhibits glycine transport (i.e., GlyT1 transporter and system-A-mediated transport), increasing glycine levels in synapses. Glycine is a positive allosteric modulator of NMDARs and acts at the same allosteric site as KYNA but with the opposite effect [96,105]. Several studies have pointed to KYNA as a ketamine-like compound due to its similar pharmacodynamics (NMDAR blockade) [106] and its ability to induce psychotic symptoms. For instance, Atlas and colleagues showed that HIV+ patients and those with psychotic symptoms had higher KYNA levels in the CSF compared to non-psychotic patients [107]. Moreover, higher KYNA levels in the CSF of 23 pairs of twins discordant for the diagnosis of schizophrenia or bipolar disorder were associated with psychotic symptoms and cluster A personality traits, typically linked to subclinical psychotic traits [108]. Given the interconnection between dopamine and glutamate systems, and with PET studies showing that stress can induce dopamine release in the striatum [109,110,111], Chiappelli and colleagues recently demonstrated that stress acutely increases salivary KYNA levels [112].

#### 5.1.2. Implications for Refractory Symptoms

As mentioned, KYNA is of particular relevance in the pathogenesis of refractory symptoms, as non-clozapine antipsychotics alleviate positive symptoms by blocking postsynaptic D2 receptors but do not target NMDAR neurotransmission, unlike clozapine, which is licensed for TRS. Indeed, several studies have highlighted the pivotal role of KYNA in TRS and refractory symptoms. Hatzimanolis and colleagues found elevated serum levels of KYNA in patients with first-episode psychosis who did not respond to first-line antipsychotics [113]. Similarly, Huang and colleagues reported that increased levels of salivary KYNA were associated with the severity of clinical symptoms in TRS [114], although other studies from the same group did not confirm these findings [114,115]. An important consideration is that TRS patients who responded to clozapine showed a lower impact of KYNA on multiple symptom domains compared to first-line responders, suggesting the greater involvement of KYNA in refractory symptoms [7]. The overexpression or overactivity of kynurenine 3-monooxygenase (KMO), the enzyme that converts KYN into KYNA due to single nucleotide polymorphisms, has been hypothesized, but the evidence remains insufficient [116]. It is important to also consider the interplay between the immune system and KP in TRS, as recently done by Chen and colleagues. The authors reported a significantly higher immune-inflammatory response compared to non-TRS patients and healthy controls. Interestingly, the QUIN/KYNA ratio interacted with immune activation in predicting the condition of TRS, further suggesting a potential role for the immune-kynurenine pathway in the pathogenesis of such condition despite pointing at a greater role for QUIN and its neurotoxicity [117].

#### 5.1.3. Implications for Cognition

Overall, the overactive KP in schizophrenia can lead to elevated levels of other metabolites, with KYNA being the most prominent at the central level [5,7,116]. KYNA likely impacts cognition primarily by modulating glutamatergic and cholinergic neurotransmission. As an antagonist at the glycine-binding site of NMDARs, KYNA reduces excitotoxicity but exacerbates glutamatergic hypofunction—a key feature of cognitive deficits in schizophrenia [21,118]. Additionally, as previously mentioned, KYNA inhibits α7 nicotinic acetylcholine receptors (α7nAChRs), impairing cognitive processes such as attention and memory [83]. Elevated KYNA levels in the CSF and postmortem brain tissues of patients with schizophrenia correlate with impairments in working memory, attention, and executive function [82,119]. Functional MRI studies link increased KYNA to reduced prefrontal activity, supporting its role in NMDAR hypofunction [105]. Preclinical studies demonstrate that increasing KYNA induces cognitive deficits in rodents, while reducing KYNA via kynurenine aminotransferase (KAT) inhibitors improves cognitive performance [118].

Inflammation further exacerbates KYNA-related cognitive dysfunction. Pro-inflammatory cytokines such as interleukin-6 (IL-6) activate kynurenine pathway enzymes, increasing KYNA synthesis and worsening cognitive deficits, particularly in patients with elevated cytokine levels [8]. Emerging therapies, including KAT inhibitors and α7nAChR agonists, show promise in targeting KYNA-associated cognitive deficits but require further validation [120,121].

In summary, KYNA contributes significantly to cognitive dysfunction in schizophrenia, and therapeutic strategies targeting KYNA metabolism hold potential for mitigating these deficits. For instance, the inhibition of kynurenine aminotransferases (KATs), which convert KYN to KYNA, enhances cognitive performance, suggesting a potential therapeutic strategy for mitigating cognitive dysfunction in psychiatric disorders. Another important metabolite is QUIN due to its ability to stimulate NMDARs, causing excitotoxicity and neurodegeneration [66,116]. Regarding cognitive functioning, KYNA has neuroprotective properties by reducing excitotoxicity, but it also impairs cholinergic and glutamatergic neurotransmission. Meanwhile, QUIN elicits detrimental effects due to its neurotoxic properties (excitotoxicity), but it sustains glutamatergic transmission. Therefore, the role of KYNA is particularly noteworthy, as it worsens positive symptoms, which are typically refractory to first-line treatments, but also affects cognition in a complex manner that remains not completely clear [7,113,114]. Overall, the role played by QUIN in neurodegenerative diseases such as Alzheimer’s points to a detrimental effect on cognition due to its neurotoxic properties, although evidence in schizophrenia is still limited [116]. Once again, considering the concomitant role of the immune system is of great importance to consider it, and Kindler and colleagues investigated the association between the KP and cognition stratifying patients in normal vs. high pro-inflammatory cytokine mRNA levels. Interestingly, only the high cytokine subgroup showed overactive KP, inferred by a higher KYN/TRP ratio and inversely related to attention and dorsolateral prefrontal cortex volume [8].

## 6. Blood–Brain Barrier: The Border Between Systemic Inflammation and Neuroinflammation

Metabolic dysfunctions cause chronic low-grade systemic inflammation, impacting multiple organs, including the CNS [122]. Despite the blood–brain barrier (BBB) having historically been considered an insurmountable barrier to systemic inflammation, recent evidence suggests associations between MetS and neurodegeneration [123], as well as between systemic inflammation and major neurological disorders [122,124]. Key drivers of this process include insulin and leptin resistance, the production of reactive oxygen species (ROS), and mitochondrial dysfunction [123]. Pro-inflammatory and dysmetabolic states can damage the BBB, allowing immune cell infiltration and pro-inflammatory marker diffusion into the brain. This infiltration can activate microglia, triggering or exacerbating neuroinflammation. The interplay between peripheral and central systems is gaining increased research attention.

### Neuroinflammation in Schizophrenia

Perinatal complications, maternal immune activation, and neonatal/childhood infections may act as early risk factors, sensitizing microglia to future “hits” from environmental risk factors in adolescence and early adulthood [13]. Indeed, postmortem studies [125] and PET imaging targeting the translocator protein (TPSO) [126] consistently report increased microglial activation, while clinical studies have identified elevated levels of cytokines, particularly interleukin 1-β (IL-1β), interleukin-6 (IL-6), and TNF-α [6]. Neuroinflammation can induce neurodegeneration, disrupt synaptic homeostasis, promote synaptic pruning, and impair myelin integrity and brain connectivity, causing long-lasting structural and functional brain alterations [127,128]. Chronic inflammation leads to brain volume loss, especially in frontal areas, due to dendritic and synaptic terminal loss, along with WM disruption. This widespread disconnection pattern [129] may partially explain schizophrenia-related symptoms and cognitive impairment [130,131,132]. Supporting this, postmortem studies show increased mRNA levels of IL-1b, IL-6, IL-8, and TNF-α in the brains of patients with schizophrenia [133,134].

## 7. Conclusions

Cognitive impairment and symptom severity in schizophrenia arise from complex and interacting determinants. Mets-induced pro-inflammatory states may influence the pathophysiology of schizophrenia through the over-activation of the KP, producing neurotoxic and neuroactive metabolites within the CNS. Pro-inflammatory cytokines elevate circulating levels of KYN, which crosses the BBB and undergoes neurotoxic transformation in the brain, contributing to disease progression. Future research should explore these interconnected mechanisms while accounting for confounders such as weight gain and antipsychotic-induced metabolic side effects, particularly from second-generation antipsychotics like clozapine, and microbiome composition. In addition, they should try to identify symptomatic/cognitive clusters more strictly related to specific patterns of KP and immune alterations with practical implications in predicting response to pharmacological treatments and cognitive remediation [135]. Overall, identifying specific patterns of association between MetS, systemic and central inflammation, and KP activation could inform tailored, integrated treatment strategies and potential precision psychiatry approaches aimed at optimizing therapeutic interventions based on individual inflammatory and metabolic profiles.

## Data Availability

No new data were created or analyzed in this study. Data sharing is not applicable to this article.
